# Obstacles and Considerations Related to Clinical Trial Research During the COVID-19 Pandemic

**DOI:** 10.3389/fmed.2020.598038

**Published:** 2020-12-23

**Authors:** Hasan Hashem, Mohammad Abufaraj, Abdelghani Tbakhi, Iyad Sultan

**Affiliations:** ^1^Division of Pediatric Hematology Oncology, Department of Pediatrics, King Hussein Cancer Center (KHCC), Amman, Jordan; ^2^Division of Urology, Department of Special Surgery, Jordan University Hospital, Amman, Jordan; ^3^Department of Cellular Therapy and Applied Genomics, King Hussein Cancer Center (KHCC), Amman, Jordan

**Keywords:** COVID-19, SARS-CoV-2, clinical research, clinical trials, ethics, misinformation

## Abstract

The response to the COVID-19 pandemic from the research and science community has been vigorous, with information being released faster than that of any other event in human history. Articles related to the virus were being rapidly published by January 2020. A small fraction of these publications comprised reports of prospective clinical trials (0.25%), and many of these trials have imparted conflicting conclusions, leading to confusion among the public and the scientific community. Additionally, the pandemic has raised many serious scientific and ethical concerns related to clinical research. In this review, we divided the conduct of clinical research trials into three steps and critically reviewed each step, along with the challenges and obstacles arising amid the ongoing crisis. The clinical research steps we reviewed include (1) clinical trial design factors such as social and scientific value, feasibility, single vs. multicenter trials, randomization, control groups, endpoints, off-label and compassionate use of medications, data analysis, and verifying the integrity of data; (2) ethical issues such as committee approvals, efficiency, virtual visits and remote monitoring, informed consent, shipping investigational products, and external monitoring and audits; and (3) publication and sharing of preprints, press releases, social media, and misinformation. The COVID-19 pandemic is adversely affecting existing clinical trials for other ailments and diseases, including cancer, with most trials being delayed or deferred. Although urgency is needed to communicate effective treatment and prevention strategies for COVID-19, research efforts should maintain the same high-quality core ethical principles that governed human subject research before the pandemic. Despite the catastrophic devastation caused by the pandemic, the adoption of more flexible, cost-effective methods of conducting clinical trials (without compromising ethical conduct, safety, or data integrity, while maintaining research efficiency) represents a potential silver lining. Streamlining clinical research will help to congruently address other important health issues, despite the ongoing COVID-19 crisis.

## Introduction

SARS-CoV-2, the causative agent of COVID-19, was identified in Wuhan, China, in early December 2019. It rapidly spread throughout China with highly efficient human-to-human transmission and has now circumnavigated the globe, becoming a worldwide pandemic. The World Health Organization (WHO) first declared it a public health emergency and subsequently a pandemic (1–3). The response to the COVID-19 pandemic by the scientific community was vigorous and with unprecedented speed. Nevertheless, the COVID-19 pandemic has disrupted all aspects of academic medical center research, raising serious concerns (4, 5).

By the time of this writing, 2145 SARS-CoV-2 studies have been registered on the ClinicalTrials.gov website (Table 1). These studies cover a wide spectrum of potential therapeutics, ranging from repurposed antibiotics, antimalarials, and antiparasitic medications to various monoclonal antibodies, targeted antiviral drugs, and stem cell therapeutics. Although the WHO has established a blueprint for performing clinical research during the pandemic, many of these studies suffer from overlapping methodologies and a distinct lack of synergy. This is particularly important because the required numbers of study subjects for these trials irrationally fluctuate, rendering some of these studies impossible to complete. The results of these studies may also later affect the design of hundreds of other studies, and ethical concerns are rising as these studies circumvent rigorous scientific standards to achieve results. Such studies and their reporting serve only to muddle facts with contradictory information and are a general disservice to clinicians practicing evidence-based medicine (EBM). Examples of contradictory information resulting from such studies include the benefit or lack thereof of incorporating corticosteroids for patients with moderately severe disease and the changing perspective of chloroquine/hydroxychloroquine efficacy and toxicity.

**Table 1:** Categories of drugs under Investigation for COVID19 Treatment or Prevention (2,145 interventional studies registered on ClinicalTrials.gov as of October 31, 2020).

**Category**	**Studies**	**Drugs**	**PubMed**	**Published clinical trials**	**Phases of studies**							**Status of studies**					
					**1**	**1/2**	**2**	**2/3**	**3**	**4**	**Others**	**Active, not recruiting**	**Not yet recruiting**	Recruiting^*^	**Completed**	**Suspended**	**Terminated/ withdrawn**
Antimalarials	196	8	1,917	27	10	3	52	26	69	21	15	15	39	85	21	11	30
Anti-inflammatory	141	24	1,399	8	10	2	33	25	45	12	14	13	30	83	17	2	4
Immune-modulators	138	4	1,320	5	20	9	51	13	17	2	26	9	18	85	16	0	2
Antivirals	122	27	869	27	3	3	56	17	30	5	8	10	30	81	9	5	6
Plasma Infusion	117	39	857	11	4	0	56	16	29	6	6	15	28	80	10	5	5
Antibiotics	83	25	615	8	4	3	20	3	21	7	25	3	26	81	5	1	5
Stem cell therapies	75	6	550	5	4	1	24	7	29	4	6	5	18	81	6	8	8
Dietary/vitamins	71	14	4,097	3	17	16	5	2	24	6	1	13	13	83	1	0	0
Others	70	17	543	93	21	21	17	1	2	1	7	7	18	82	5	0	1
Antiparasitic	66	5	114	1	1	2	23	13	15	4	8	2	23	81	8	0	0
Antibodies	64	19	608	4	2	2	13	7	21	13	6	2	19	82	1	0	2
Anticoagulant	55	31	5,388	4	4	8	20	2	9	7	5	5	11	81	2	1	1
Steroids	51	7	761	9	0	1	13	6	15	9	7	2	10	80	6	1	4
Cardiovascular/antihypertensive	49	25	648	1	1	1	26	10	8	2	1	7	10	80	4	2	2
Vaccines	43	15	182	14	2	3	19	6	6	4	3	2	6	80	2	1	4
Targeted therapies	40	7	119	3	3	4	20	3	8	1	1	5	14	80	0	0	1
Cytokines	32	5	706	17	2	1	15	1	6	5	2	2	8	83	5	1	0
ACE receptor targeted	24	11	789	1	3	0	8	3	3	5	2	0	8	81	2	1	1
Neurologic/anesthetic	21	13	116	3	2	0	7	2	3	3	4	3	8	80	0	0	1
Hormonal (other than steroids)	20	12	1,095	12	3	1	8	2	4	2	0	2	6	80	0	0	1
Traditional/herbal	13	9	746	10	0	2	2	5	2	0	2	0	2	80	1	2	0

*Data extracted from ClinicalTrials.gov on October 31, 2020; PubMed search (October 31, 2020) shows hits of drugs in each category in combinations with the following search world (COVID OR COVID-19 OR COVID19 OR SARS-COV-2 OR SARS-COV2); Rows may not add up to the expected total due to some missing or unknown (e.g., status “No longer available” or “Active Not Recruiting”). Columns may not add up to the expected total number due to the overlap in some drugs (e.g., targeted therapies and antibodies) and inability to categorize some studies (e.g., studies of medical devices)*.

* *Recruiting studies include studies recruiting by invitation*.

## Summary of Research Published During the COVID-19 Pandemic

A Medline search using the keywords COVID19, COVID-19, and SARS-CoV-2 identified all citations until October 31, 2020. Citations were then categorized according to the type of reference, month of publication, and language. The same keywords were used to search for citations that also included drugs in each category listed in Table 1. An automated search method using R (Version 4.0.2) and Easy PubMed package (v 2.13) was used to automatically retrieve citations for different categories.

**Figure 1:**
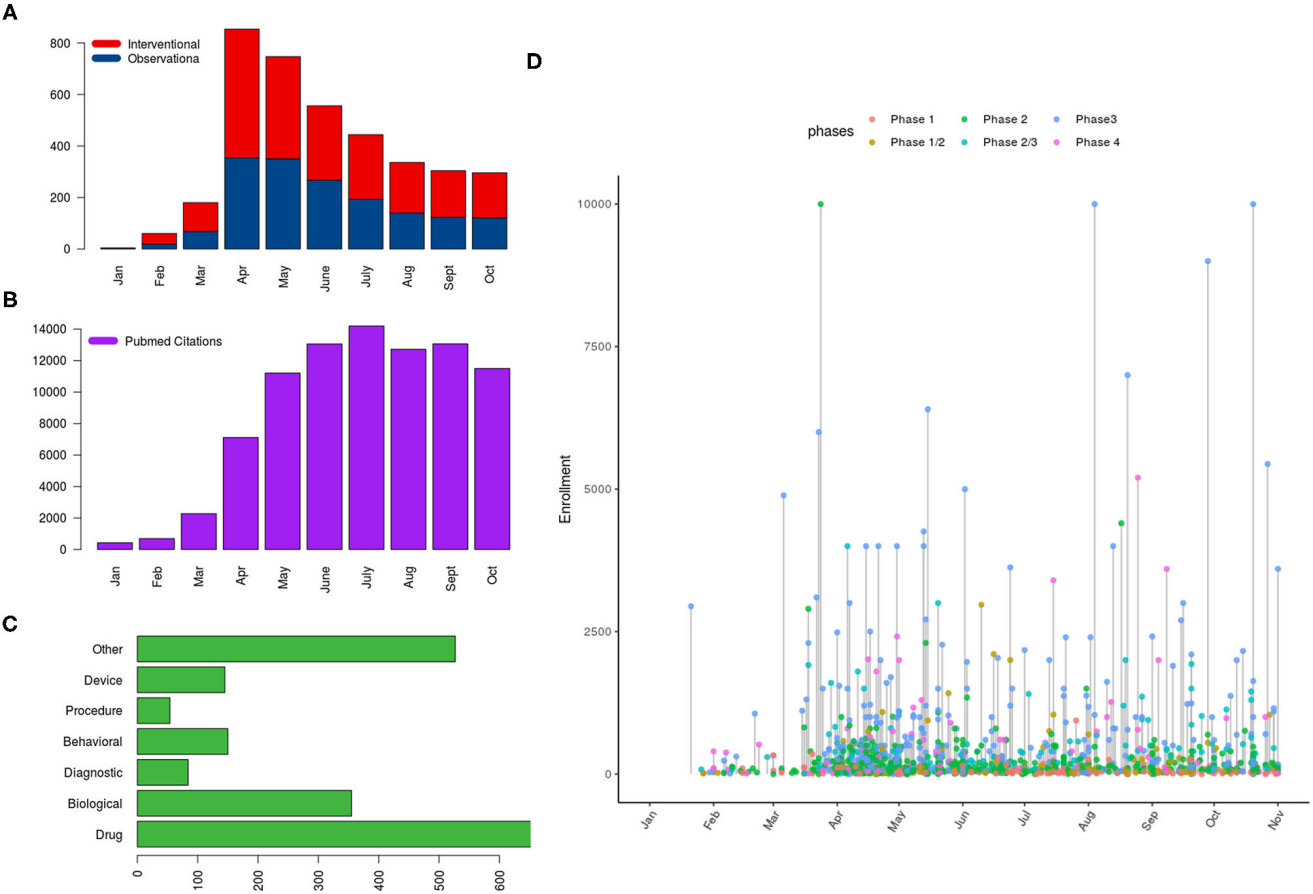
Distribution of clinical trials registered on ClinicalTrials.gov and PubMed citations referring to COVID-19 as of October 31, 2020; the panels show **(A)** the distribution of studies according to type and month of posting, **(B)** number of PubMed citations per month, **(C)** the type of intervention in interventional trials, and **(D)** a lollipop graph showing the anticipated number of subjects to be enrolled on interventional trials with the horizontal axis indicating the date of first posting of studies (year 2020) and the *y* axis indicating the required number of subjects to be enrolled (capped at 10,000); color of the points indicates the phase of trial.

**Figure 2:**
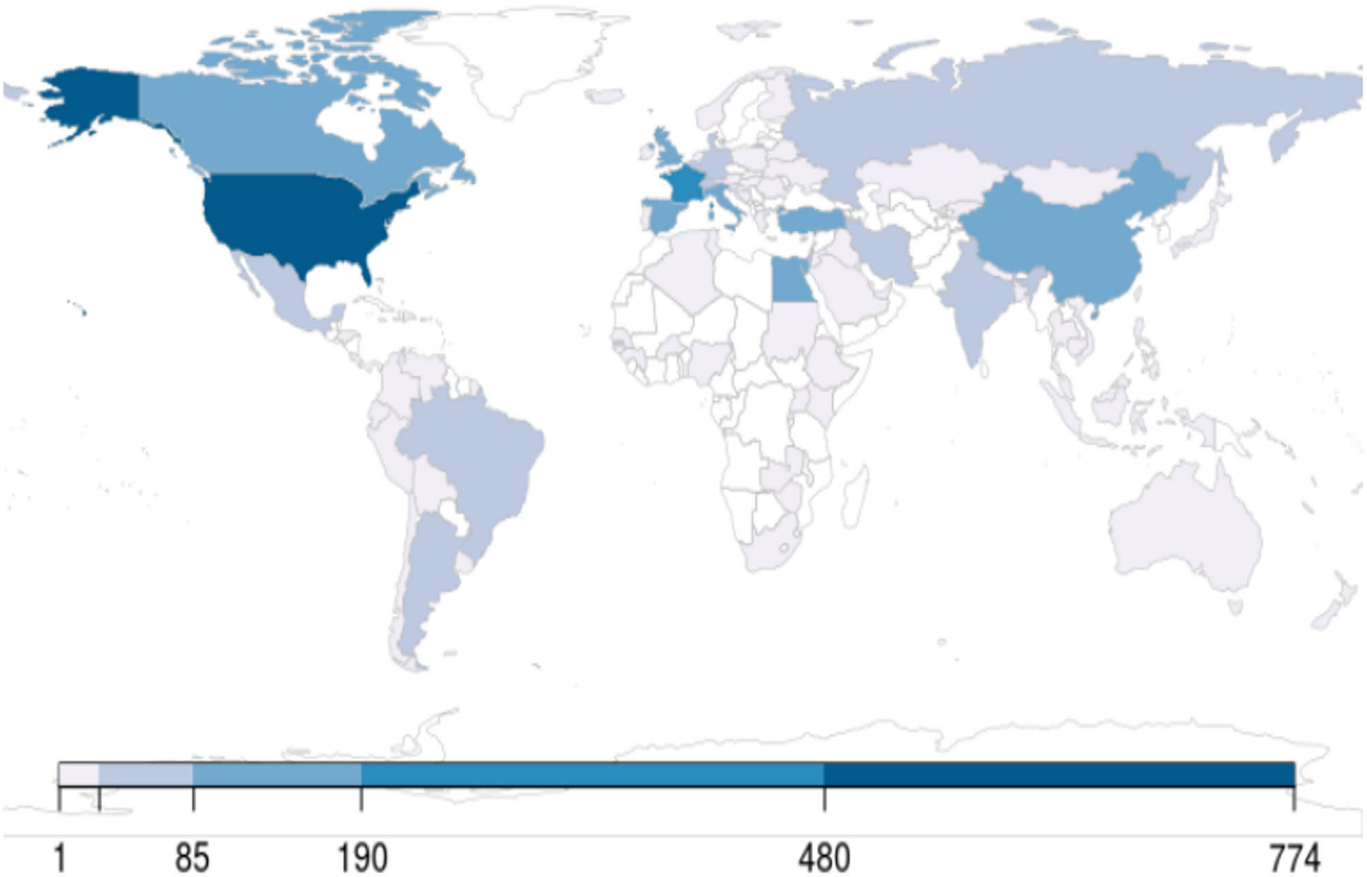
World map showing the distribution of clinical trials, counted per the primary institution listed on ClinicalTrials.gov as of October 31, 2020.

By October 31, 2020, 71,004 articles were cataloged by the National Library of Medicine. The number of articles increased sharply since January 2020: 428 published in January, 689 published in February, 2269 published in March, 7109 published in April, 11,206 published in May, 13,056 published in June, 14,199 published in July, 12,717 published in August, 13,061 published in September, and 11,495 published in October. The majority (95%) of articles were written in English, followed by Chinese and French (1% each). Only 180 (0.25%) studies out of 71,004 comprised clinical trials including randomized controlled trials (RCTs). A small proportion of publications also reported observational studies (*n* = 559), systematic reviews (*n* = 1072), and meta-analyses (*n* = 349). Editorials and letters represented nearly one-fourth of COVID-19 publications (*n* = 16,561, 23%) (Figure 1). As of October 31, 2020, The United States published the highest number of studies, followed by France and China (Figure 2).

## Examples of Major Flaws and Misinformation Published During the COVID-19 Pandemic

Although the pressure and urgency for conducting COVID-19 research abounds during this worldwide crisis, this should not preclude scientific principles and ethics (6). Pandemics raise difficult scientific and ethical questions for research in this climate. Therefore, understanding what ethical concerns remain the same and what differs is important for conducting clinical trials during pandemics. For example, the first case report of presymptomatic transmission published in the *New England Journal of Medicine* (*NEJM*) was based on incorrect information because the researchers did not interview the patient, believing her to be asymptomatic during the period in which she exposed others to the virus. However, when German investigators subsequently interviewed her, she reported having symptoms at the time of transmission (7). Additionally, some patient experiences were reported in more than one publication, as described by the editors of the *Journal of the American Medical Association* (8). In a study published in *NEJM* describing critically ill patients who received remdesivir, the time to clinical improvement was calculated as a time event without considering death as a competing risk. This inflated public belief of the drug's benefits because deceased patients do not have an equal chance of improvement and thus cannot be censored (9).

**Figure 3:**
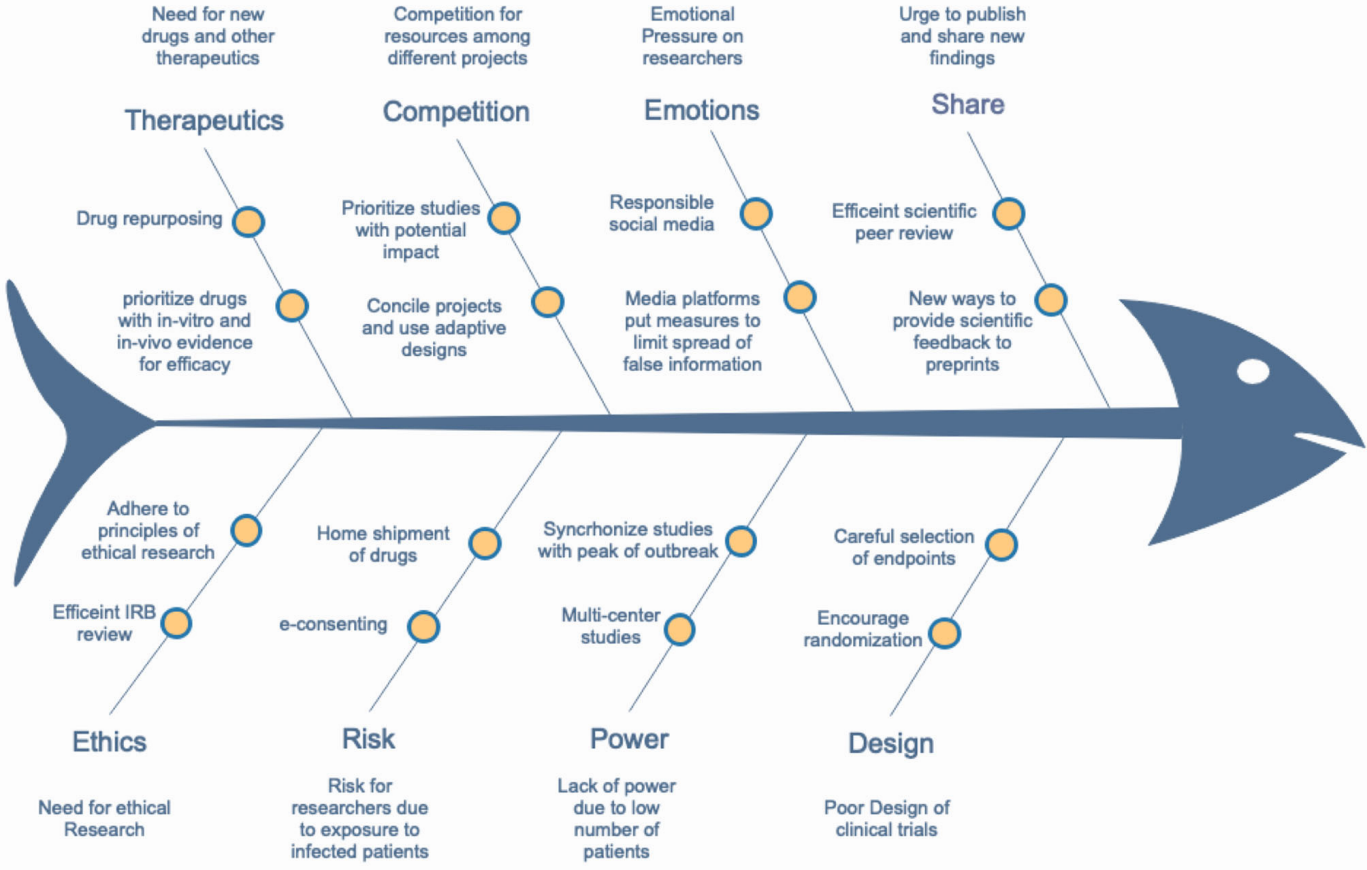
A fishbone diagram showing the main obstacles for conducting efficient COVID-19-related research and some suggested solutions.

In the following sections, we list and dissect the steps of conducting clinical research in terms of challenges and obstacles that researchers experience and propose solutions to achieve ethically adherent and scientifically sound research (Figure 3).

## Designing Scientifically Solid Research

### Scientific and Social Value

All research or clinical trials should embody certain concepts and principles to be considered informative and valuable. Research generally begins with a hypothesis. The aim of this hypothesis and its testing should be important, clinically meaningful, and of value to study participants. The interventions selected for testing should consist of the most promising therapies, as determined by existing data. The value of clinical trials depends on the quality of information produced and the relevance of the data to address public health needs. Nevertheless, there are many less-developed countries that do not have well-prepared medical infrastructure and little or no experience in conducting trials. Moreover, there is considerable heterogeneity across countries and even within each country, when it comes to health care systems. This may result in some differences in many aspects starting from the review process itself to all the other steps of conducting trials such as data monitoring and patient safety. Accordingly, these regional differences should be closely monitored when conducting clinical trials. Clinical trial design should be rigorous and analyzed with full integrity. The knowledge gained should be reported completely, promptly, and consistently. These trials should meet all regulatory standards and conducted in an effective and safe manner. Sound scientific research principles should not be compromised even during pandemics (10, 11).

### Resource Allocation

As the COVID-19 pandemic unfolds, preparedness programs are taking precedence over non-clinical activities deemed non-urgent. Research is a key aspect of responding to pandemics, yet it should never impede response efforts, such as maintaining personnel, equipment, and facilities for treating patients (12). Health care systems are frequently overwhelmed during pandemics because all resources are allocated and diverted to quell the pandemic. All countries share the common constraint of finite budgets and resources for combating pandemics, which is particularly true for the current COVID-19 pandemic (13). Such restricted resources are challenging for multiple steps of conducting clinical research. For example, study feasibility may be affected, leading to a sense that the study may never be completed. For that reason, researchers, sponsors, and regulators must make exceptional efforts to cooperate and collaborate to concentrate resources in the most efficient way while concomitantly ensuring that the standards of scientifically sound research are not relaxed (14). This may be accomplished by testing multiple interventions in collaborative multi-institutional trials. Nevertheless, there are many challenges in multicenter large-scale clinical trials. First, complex protocols will increase pressure on the coordinating center to maintain oversight and avoid deviations. Second, lack of workflow standardization across research sites. Third, data collection and protocol adherence could be challenging due to differences in laws and regulations among different countries. Collaborative efforts among national policy makers, the pharmaceutical industry, opinion leaders, patient advocacy groups, and regulatory agencies are imperative for containing the pandemic because of their oversight roles, which should be used to expedite trials that meet all of the standard core ethical and scientific requirements but also minimize and prevent duplicated and underpowered studies.

### Drug Repurposing

Drug repurposing is an attractive strategy for treating a novel disease because it offers lower costs and reduced time to reach the market. This strategy alleviates some clinical trial steps, especially those concerning the strenuous diligence and time required for phase 1 and 2 trials (15). Because the safety profiles of repurposed drugs are established, using previously existing therapeutic agents designed to treat other diseases and pathologies, especially those similar to SARS-CoV-2/COVID-19, is a particularly appealing approach (16, 17). Moreover, this approach may be the only practical method for establishing a rapid response to an emerging pandemic. Indeed, existing pharmaceutical supply chains are available for formulation and distribution.

### Evidence vs. Emotional-Based Medicine

EBM is not and should never be emotion-based medicine. “Listening to your gut,” administering unsubstantiated treatments in a panic response, and conducting hasty science are regressive approaches. The unprecedented speed of concept-to-implementation RCTs in only a few weeks provides proof of concept that properly conducted RCTs can be promptly initiated in the middle of a pandemic. Abandoning sound scientific principles in the face of pandemic simply because we are overwhelmed is clearly unacceptable (18).

## Institutional Review Board and Ethical Approval

### Ethics in Research During the COVID-19 Pandemic

Planning and conducting clinical research during pandemics elicit a number of ethical issues that must be addressed. To this end, some stakeholders debated whether it is ethical to conduct research at all in the midst of a pandemic. Some were skeptical of activities that may draw efforts away from the mission of providing clinical care to patients affected by the pandemic. However, some argued that the pandemic presents the best opportunity to conduct COVID-19 clinical research. Indeed, the WHO Research Ethics Review Committee stated that conducting research is an ethical obligation. Despite the sense of urgency elicited by the pandemic, research is still subject to the same core ethical principles that govern research on human subjects. Specifically, clinical research must minimize harm by saving lives and ensuring that informed consent is always obtained, despite the pandemic, while ensuring efficient use of resources (19). However, a paltry amount of studies focusing on ethical guidance for conducting research during pandemics are published (20–23). Nevertheless, the way we currently conduct research must be adaptable and evolve as the current pandemic unfolds because it can provide us with a new understanding and discovery of methods that can make conducting research faster, safer, and more efficient. Maintaining ethics in research is imperative to providing answers for questions in which no black-and-white answers are available. One such question is how to ethically allocate scarce resources for research when health care systems are stretched beyond capacity. Another such question is how to ethically balance the public health resources needed to combat the pandemic with those needed by research designed to find potential remedies for the same pandemic (24).

### Institutional Review Board Efficiency

Thousands of clinical trials were registered in the first few months after COVID-19 was declared a pandemic. If ethics committees cannot review such a large number of clinical trials and ensure that they maintain a high standard, many high-risk and low-benefit drugs may potentially be used to treat patients with COVID-19. Not only will these patients be at risk for unknown complications but valuable resources may also be unallocated for more meaningful research. The ethical review for COVID-19 research at this time occurs under exceptional circumstances. Institutional review boards (IRBs) should particularly consider such issues as strict inclusion and exclusion criteria, participant compensation, and clearly defined risks of the trial to vulnerable patients (25). Moreover, IRBs must ensure that the standard of ethical review is not relaxed (26). To improve IRB expediency during pandemics, pre-study documents should be available and generally easy to complete as quickly as possible. Such documents include signed protocols by principal investigators, financial disclosure forms, conflict of interest disclosure forms, letters of agreement with sponsors, and informed consent forms. Template case report forms (CRFs) should be made available for modification and online entry. IRBs should be continuously informed of research progress. Notifying IRBs about form modifications may also help to expedite the review process.

### Virtual Visits and Remote Monitoring

Travel bans, quarantines, and stay-at-home measures have been implemented to variable degrees throughout the world. Moreover, the risk of transmission of infection not only for participants (if they are healthy) but also for research staff who should be aware of the added risk of infection during in-person visits is an important consideration during pandemics. This introduces limitations on scheduled study assessments and procedures for patients. Therefore, careful risk assessments must be performed before applying for IRB approval to establish in-person visit purpose, frequency, and extent of monitoring needed for proposed clinical trials (27, 28). To mitigate the likelihood of infection, remote monitoring in the form of telephone and/or video visits is strongly recommended but should be limited to essential core data and kept to a minimal frequency to avoid unnecessary burden on the investigator and trial team. These essential data include screening for inclusion and exclusion criteria, investigational drug doses and dose regimens, and serious adverse events. Using patient local facilities for laboratory investigations and imaging are also alternative approaches for regular study assessments. However, such modifications depend on the type of research, as some studies require frequent monitoring and require face-to-face encounters (29, 30).

### Shipments of Investigational Products

To ensure the safety and well-being of participants and to ensure the continuation of clinical trials according to their protocols during the COVID-19 pandemic, it may be necessary to send investigational drugs directly to trial participants. Pharmacovigilance remains of paramount importance to ensure the security, accountability, traceability, and compliance of participant-administered investigational drugs. To maintain patient privacy and data confidentiality, delivery of investigational products directly from trial sites to patients may be necessary. Shipments should occur in a manner that allows tracking of both transport and delivery, and participants should acknowledge receipt of shipments. Written instructions on the storage and use of the investigational drugs should be provided to participants. Moreover, documentation of all communication between providers and patients and instructions remains vital (29).

### Informed Consent

Since the medical guidelines established by the Nuremberg Code and later the Declaration of Helsinki were introduced, informed consent became a common and fundamental part of clinical research. The quality of the consent process greatly depends on the time constraints of the procedures. Obtaining informed consent is usually performed with paper forms explaining the research purpose, procedures, and potential adverse effects, which are signed by participants. During pandemics, researchers must consider the risk of transmission of infection through paperwork. Because data acquisition, capture, and storage are often performed electronically, electronic acquisition of informed consent is logical. Verbally attained consent for patients under quarantine can be obtained first in the presence of a witness followed by written consent when the participants are released from quarantine. An alternative approach to minimizing the risk of infection while maintaining all principles of informed consent is through virtual e-consents (31). However, the electronic system for virtual e-consents must include a method to verify identity. Study personnel should also ensure that the information presented to participants is understandable in a language they comprehend. This may be addressed by a checkbox (i.e., “I understand and agree”). Study personnel may help navigate the consent process by clicking on links for the participants. Study participants should also be provided with enough time to meaningfully complete the informed consent process. This may be challenging for sick and critically ill patients; therefore, a surrogate decision maker or legally authorized representative can obtain consent. Ideally, a uniformly accepted procedure should be adopted for all investigators performing research with critically ill subjects (32–34).

### External Monitoring/Audits

Oversight responsibilities should be maintained during pandemics to ensure the quality of the research. Temporary alternatives for external monitoring should take into account appropriate oversight and site capacity. Such alternatives may include postponing of on-site monitoring visits, extending the period between visits, and implementing video or phone visits supplemented with centralized monitoring and review. Audits should be postponed and, when conducted, should follow social distancing roles. As the pandemic ends, robust visits and monitoring should return to the pre-pandemic processes. We acknowledge that the COVID-19 pandemic will most likely introduce protocol deviations; these deviations should be managed according to standard procedures in a manner that is in the best interest of the participants without exposing them to unnecessary risks (35).

## Clinical Trial Design/Conduct

### Single vs. Multi-Center Trials

The urgency of the international response to the COVID-19 pandemic has challenged research coordination and collaboration, resulting in hundreds of independent efforts to test various interventions (13). To achieve rapid yet scientifically sound results, research duplications and competition for recruitment should be avoided (14). Nevertheless, data collection in multicenter trials is challenging. By engaging multiple sites, timely insights into important design and feasibility issues of the recruitment rate and protocol adherence can be acquired. Data collection that is internet-based may facilitate these scenarios. The COVID-19 pandemic has underscored the need for trust in science and global collaboration. Many national regulatory authorities have set up streamlined and fast-track clinical trial approval processes. However, the lack of harmonization between national regulations is slowing down the implementation of international clinical trials. Governments and key regulatory authorities are encouraged to seize the opportunity provided by the current exceptional situation to significantly advance the international harmonization of multiple aspects of clinical trial regulations. There are few examples of international efforts such as working with the International Council for Harmonization (ICH), which has developed a number of guidelines such as MedDRA (Medical Dictionary for Regulatory Activities) for the harmonization of the technical requirements for pharmaceutical products and could facilitate discussion on regulatory standardization. Another example is CARE (Corona Accelerated R&D in Europe), a new consortium supported by the Innovative Medicines Initiative (IMI) public–private partnership announced to accelerate the discovery and development of urgently needed medicines to treat COVID-19.

### Large vs. Small Trials

Adequately powered trials are essential for making important discoveries. A study that enrolls thousands of patients can answer vital questions with confidence, such as whether or not COVID-19 is treatable. However, these studies involve very complex logistics and are consequently very expensive, reducing the ability to screen an adequate number of drugs. If a drug is truly capable of treating COVID-19, this should be evident in a small sample. Endpoints should be designed to capture this difference. For example, achieving a 50% reduction in the time to clinical improvement requires a smaller cohort of patients who need to be treated (NNT) than does a drug achieving a 20% reduction in the time to clinical improvement. The former is more clinically relevant, but the latter is more sensitive and is more likely to avoid premature withdrawal. The NNT cost should be balanced to the available resources and number of agents to be tested. An adaptive approach that permits dynamic changes in the NNT and endpoints according to interim analysis results is being used more commonly during the pandemic (36, 37).

### Feasibility

Studies must be feasible and thereby designed so that they can be completed within a time frame that the findings are still relevant. Priority should be given to interventions that reflect the specific needs of the patient population and are readily implementable. For patients in low-income countries, interventions should be affordable and rapidly available. During a pandemic, greater flexibility is needed for conducting clinical trials. A move toward decentralized clinical trials conducted across satellite sites may improve the adaptability of such trials (38, 39). In decentralized clinical trial models, data can be collected at remote locations *via* modern virtual methods. However, barriers and challenges to this model include a greater reliance on data security and increased complexity in supply chain logistics. The solution to these challenges is a hybrid model incorporating decentralized components only during times of crisis, but a greater degree of risk sharing than is currently acceptable is necessary.

### Randomization

COVID-19 trials should have a rigorous design; they should be adequately powered and well-designed to generate clinically meaningful data. RCTs are the gold standard for providing efficacy data (18). During pandemics, the temptation to make unproven therapies widely available and not waiting for rigorous clinical trial data to be generated is understandable (25, 40). However, RCTs can be conducted quite rapidly. Thousands of new patients with COVID-19 seek care each day worldwide; therefore, patient accrual requirements, an often rate-limiting step of clinical trials, can be met quickly for COVID-19 clinical trials. However, the sense of urgency to discover efficacious treatments for COVID-19 should not circumvent high standards of research because this could prove detrimental to their quality. The moral mission of research remains the same—to reduce uncertainty and enable caregivers and health care systems to address individual and public health matters. Randomization between low- and high-dose drug treatment arms or between short and long drug durations is only useful after the investigational drug is found to be more efficacious than the standard of care. The rush to offer unproven treatments outside of well-designed clinical trials undermines high-quality science and condemns us to repeat age-old errors.

Many factors can contribute to the fallacy of research exceptionalism (10). First, some evidence, even if flawed, may be preferable to those seeking immediate treatments than is expanding resources on more demanding studies whose benefits will only materialize later. The rapid results generated by hasty research are generally less adherent to the established protocols and quality controls required to produce sound science. Second, some may view that randomizations and placebo comparators conflict with clinician care obligations in urgent conditions. Third, researchers and sponsors may be assumed to be free to exercise broad discretion over trial design. However, most small non-controlled or non-randomized studies are arguably built upon preclinical research findings that are often not confirmed in subsequent well-designed trials. The case for and against hydroxychloroquine is a notable example of this (41). It is important for researchers to realize that every patient treated in an uncontrolled trial is someone being subjected to experimentation without the possibility of contributing to the body of scientific knowledge. Adaptive-designed RCTs should be prioritized during the COVID-19 pandemic and future pandemics. Such RCTs permit investigators to accept or reject multiple experimental therapies throughout the trial, dropping those showing the weakest efficacy and adding new promising treatments, while remaining adequately powered (36, 37, 42).

### Off Label, Compassionate Use, and Historical Controls

During the Ebola outbreak in 2014, numerous therapies were tested. Ultimately, however, none were found to be efficacious. Because nearly all of these studies comprised single-arm trials with no concurrent controls, no definitive conclusions emerged (43). The world is now facing a similar situation with the COVID-19 pandemic, with no proven therapies materializing after 6 months from the start of the pandemic. Administering unproven drugs as a last resort incorrectly assumes that the chance of it benefiting the ill is higher than the chance of harming them. In the absence of a control group, it is impossible to know whether patients are benefited or harmed. Furthermore, determining whether adverse effects occurring in patients are caused by the investigational drug or the disease is irresolvable (44). Other methods of comparison, such as historical control data, are unlikely to produce reliable results because supportive care approaches frequently evolve. A common but untrue interpretation of compassionate and off-label drug administration is that if patients die, it is of their disease, but if they survive, it is because of the drug. Discovering new drugs while simultaneously ensuring that they will most likely help to relieve disease symptoms over than of alternatives is imperative; otherwise, therapies for future coronavirus pandemics are not guaranteed, risking another worldwide standstill in the future (45, 46).

### Endpoints

Surrogate measures are not intrinsically beneficial to patients but are designed to be easier and faster to measure than clinically meaningful outcomes. Surrogate endpoints trade the advantage of reducing the time needed to conduct clinical trials for the disadvantage of treatment effect uncertainty. However, during the tumultuous events unfolding during pandemics, when pressure constantly runs high, does this same strategy still hold true? Whether this trade-off is beneficial or detrimental to patients deserves further scrutiny. French investigators were the first to report promising hydroxychloroquine data, although their study was underpowered and six patients were removed from analysis because of unfavorable outcomes (47). Their erroneous positive findings were due to using surrogate measures, i.e., SARS-CoV-2 clearance. Determining the extent in which randomization should have in trials of new interventions is an important consideration. It is also important to consider the endpoints being measured. For example, survival or 28-day morality would be useful endpoints for clinical trials of ventilated patients who have high mortality rates. In contrast, seven-category ordinal scales, which are recommended by the WHO, may be more useful primary endpoints for trials of mild-to-moderate cases because these patients have a much lower risk of death. Moreover, seven-category ordinal scales may minimize potential bias between different trials and sites for their definitions of severity (48).

## Data Analysis and Integrity

In any clinical trial, information should be collected, recorded, and handled in a way that allows for accurate reporting, interpretation, and verification. Trial success depends on the quality and management of the collected data. Subject privacy should be protected by identification numbers or other methods. Patient folders should contain completed informed consent forms, screening sheets clarifying inclusion and exclusion criteria, patient CRFs, laboratory values, and a record of all communication with the subject. Data safety monitoring boards with relevant clinical expertise, completely independent of the investigators, should be available to evaluate interim data to ensure that participants are not exposed to additional risks (35). During the COVID-19 pandemic, participants have been hesitant of going to hospitals. Therefore, alternative methods, such as telehealth-mediated patient visits, are encouraged to obtain data. These designs should be pre-specified in protocols, prospectively registered, and analyzed accordingly.

## Publication and Sharing

### Peer Review and Preprints

Researchers are ethically obligated to share information as soon as it is quality controlled for release (i.e., peer-reviewed). This may add pressure to the peer-review process to increase efficiency during pandemics. Because reviewers are a scarce resource, especially during pandemics, this can lead to an influx of low-quality publications. Moreover, depositing positive findings to preprint servers earlier than negative findings can introduce bias and may be misleading. Although preprints may expedite communication of notable findings, they also entail certain risks. Many preprints are later rejected or changed to state different conclusions that were initially stated. The publication process must adhere to the principles of publication ethics to promote integrity, accuracy, and value of scholarly publications. These principles are as follows: (1) ensure scientific accuracy and validity through peer review, (2) provide social value, (3) protect participants and affected communities by ensuring that reviewers respect and maintain patient confidentiality and ethics, (4) disclose conflicts of interest and limitations of the data, and (5) hold researchers and journal editors accountable for published data (49, 50). The pressure to publish COVID-19-related articles has led to fast-tracking the peer-review and publication process, resulting in six- to eightfold faster reviews and subsequent online publications than before the COVID-19 pandemic (51). Because the review process is often criticized as a lengthy process that is less efficient than the needs of the scientific community before the pandemic, lessons from this experience should be extended after the pandemic ends.

### Social Media, Press Releases, and Misinformation

At the time of this writing, many dubious COVID-19 cures and miracle remedies have spread across social media, reaching vast audiences every day. Social media and online sites are the primary platforms from which false, inaccurate, and misleading information is disseminated because they facilitate rapid and large-scale sharing with little to no adherence to the traditional mechanisms of quality control and gate-keeping outside of the scientific community (52, 53). Misinformation, in which misleading stories are circulated generally in good faith, can propagate outright falsehoods. The demonization of vaccinations on the basis of shoddy and untrue data is a well-known example of misconstrued medical and health care information, culminating in the “anti-vax” movement (54). Therefore, it is not surprising that the COVID-19 pandemic has also been inundated with misinformation. Despite the lack of an effective cure for COVID-19 and thousands of clinical trials registered on ClinicalTrials.gov, misleading news of many potential therapies continues to spread on social media, building hype toward them without acknowledging that many trials will most likely result in negative findings and provide no use toward ending the pandemic. The WHO warned in February 2020 that the COVID-19 pandemic is coupled to an infodemic, i.e., an overabundance of information and misinformation masquerading as truth. The consequences of such infodemics are the spread of uncertainty, fear, and anxiety (55, 56). To mitigate the harm caused by the infodemic, the WHO created a section on its website devoted to myth-busting and debunking false information. As of August 2020, the WHO has been publishing daily reports to provide the population with reliable data. Moreover, search engines such as Google and social media platforms such as Facebook, Twitter, and YouTube have established measures to both limit the spread of false information and direct users to reliable sources (57).

## Effect of the COVID-19 Pandemic on Non-COVID-19 Research (Cancer Research as an Example)

The complexity of cancer research has been further complicated by the COVID-19 pandemic. COVID-19 has interrupted the launching of new clinical trials because of reduced resources (29, 58). Many patients were enrolled in clinical trials before the pandemic, and as the pandemic progressed, investigators were forced to limit patient visits and constrain their research to essential laboratory studies, causing delays in data collection and reporting (59). The COVID-19 pandemic is halting subject recruitment and hampering the speed and quality of data collection and analysis. To minimize the impact of the pandemic on research, clinical trials investigating potentially life-saving drugs should be prioritized. Investigators conducting clinical trials during the pandemic must be wary because increased protocol deviations can be expected, potentially affecting general patient safety due to missing or late reporting of adverse events (24). The Centers for Disease Control and Prevention and National Institutes of Health have both released guidelines for continuing research during the COVID-19 pandemic (60, 61). Trial sponsors should expect missed follow-ups and report them as deviations. Establishing contingency plans and maintaining sponsor and contract research organization alignment are some of the key issues for continuing cancer research (62–64).

## Conclusion

The international scientific community must review and self-criticize its response to the COVID-19 pandemic. With more than 40 million people affected and 1 million deaths, efforts should not concentrate on any single aspect of conducting clinical trials but should rely on high-quality standards to demonstrate which therapeutic strategies are the most beneficial for patients. Although we cannot reliably predict which intervention will be most effective for treating COVID-19, well-designed, unbiased clinical trials are necessary to elucidate these interventions. Genuine knowledge can only be gained through objective scientific methods rather than personal or emotionally driven methods, such as mere conjecture or empiricism. Adapting more efficient and cost-effective methods for conducting clinical trials, without compromising ethical conduct, safety, or data integrity, should be the lesson learned from this catastrophe. We will repeat these mistakes in the next pandemic if we do not implement what we have learned in our future research endeavors.

## Author Contributions

HH and IS designed the study, reviewed the literature, and wrote the manuscript. MA and AT wrote the manuscript. All authors approved the final manuscript.

## Conflict of Interest

The authors declare that the research was conducted in the absence of any commercial or financial relationships that could be construed as a potential conflict of interest.
